# *Phlebotomus* (*Adlerius*) *simici* NITZULESCU, 1931: first record in Austria and phylogenetic relationship with other *Adlerius* species

**DOI:** 10.1186/s13071-020-04482-8

**Published:** 2021-01-06

**Authors:** Edwin Kniha, Vít Dvořák, Markus Milchram, Adelheid G. Obwaller, Martina Köhsler, Wolfgang Poeppl, Maria Antoniou, Alexandra Chaskopoulou, Lusine Paronyan, Jovana Stefanovski, Gerhard Mooseder, Petr Volf, Julia Walochnik

**Affiliations:** 1 Institute of Specific Prophylaxis and Tropical Medicine, Center for Pathophysiology, Infectiology and Immunology, Medical University of Vienna, Vienna, Austria; 2 Department of Parasitology, Faculty of Science, Charles University, Prague, Czech Republic; 3 Department of Integrative Biology and Biodiversity Research, Institute of Zoology, University of Natural Resources and Life Sciences, Vienna, Austria; 4 Division of Science, Research and Development, Federal Ministry of Defence, Vienna, Austria; 5 Department of Dermatology and Tropical Medicine, Military Medical Cluster East, Austrian Armed Forces, Vienna, Austria; 6 Laboratory of Clinical Bacteriology, Parasitology, Zoonoses and Geographical Medicine, Faculty of Medicine, University of Crete, Heraklion, Greece; 7 European Biological Control Laboratory, US Department of Agriculture–Agricultural Research Service (USDA–ARS), Thessaloniki, Greece; 8 Vector Borne and Parasitic Diseases Epidemiology Department, National Center for Disease Control and Prevention, Ministry of Health, Yerevan, Armenia; 9 Department of Parasitology and Parasitic Diseases, Faculty of Veterinary Medicine, Saints Cyril and Methodius University, Skopje, North Macedonia

**Keywords:** Phlebotomine sand fly, Central europe, *Adlerius*, *Leishmania infantum*, Refugial area

## Abstract

**Background:**

Phlebotomine sand flies are the principal vectors of *Leishmania* spp. (Kinetoplastida: Trypanosomatidae). Information on sand flies in Central Europe is scarce and, to date, in Austria, only *Phlebotomus mascittii* has been recorded. In 2018 and 2019, entomological surveys were conducted in Austria with the aim to further clarify sand fly distribution and species composition.

**Results:**

In 2019, a *Ph. simici* specimen was trapped in Austria for the first time. Analyses of two commonly used marker genes, cytochrome *c* oxidase I (*coxI*) and cytochrome* b* (*cytb*), revealed high sequence identity with *Ph. simici* specimens from North Macedonia and Greece. Phylogenetic analyses showed high intraspecific distances within *Ph. simici*, thereby dividing this species into three lineages: one each from Europe, Turkey and Israel. Low interspecific distances between *Ph. simici*, *Ph. brevis* and an as yet unidentified *Adlerius* sp. from Turkey and Armenia highlight how challenging molecular identification within the *Adlerius* complex can be, even when standard marker genes are applied.

**Conclusion:**

To our knowledge, this study reports the first finding of *Ph. simici* in Austria, representing the northernmost recording of this species to date. Moreover, it reveals valuable insights into the phylogenetic relationships among species within the subgenus *Adlerius*. *Phlebotomus simici* is a suspected vector of *L. infantum* and therefore of medical and veterinary importance. Potential sand fly expansion in Central Europe due to climatic change and the increasing import of *Leishmania*-infected dogs from endemic areas support the need for further studies on sand fly distribution in Austria and Central Europe in general.

**Graphic abstract:**

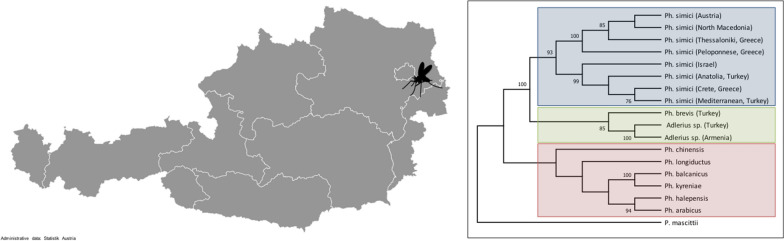

## Introduction

Phlebotomine sand flies (Diptera: Psychodidae: Phlebotominae) are small hematophagous insects and vectors of the protozoan parasites *Leishmania* spp., the causative agents of leishmaniasis. In Europe, sand flies were long considered to be primarily present in the Mediterranean basin, where both visceral and cutaneous leishmaniasis are endemic [1]. The occurrence of sand flies north of the Alps was overlooked until *Phlebotomus mascittii* GRASSI, 1908, and *Phlebotomus perniciosus* NEWSTEAD, 1911, were found in Germany in 1999 and 2001, respectively [2, 3]. Shortly thereafter, the presence of *Ph. mascittii* was reported in northern France and Belgium [4]. Surveys carried out 2010–2013 also revealed stable *Ph. mascittii* populations in four federal states of eastern Austria [5–7], and a singular specimen was trapped in western Slovakia in 2016 [8]. In addition to records of *Ph. mascittii*, stable populations of *Phlebotomus neglectus* TONNOIR, 1921, were reported in Hungary [9, 10].

Sand flies in Central Europe are assumed to be remnants of post-glacial recolonization events from Mediterranean refugial areas that have survived in small, microclimatic areas [11]. This hypothesis is further supported by a model of the potential distribution of Mediterranean sand fly species up to northern European countries, including the UK, during the Holocene optimum approximately 6000 years ago [11]. *Phlebotomus mascittii*, an unproven but suspected vector for *Leishmania* spp., has been assumed to be the only sand fly species in Austria; however, considering the absence of a geographic barrier between Hungary, Slovenia and eastern Austria, the possible occurrence of other species via prospective dispersal to Austria is likely. Reports of suspected autochthonous leishmaniasis cases in Austria highlight the necessity for further research [12, 13].

Entomological surveys were conducted in Austria in 2018 and 2019 with the aim to update available knowledge on species composition and distribution of sand flies in Austria. The identification of caught specimens to the species level was achieved through a combination of morphological and molecular approaches, and their phylogenetic status was also evaluated. Here we report the findings of these surveys in relation to a newly reported species.

## Material and methods

### Entomological survey

Entomological sand fly surveys were conducted in six federal states of Austria, in July and August 2018 and 2019. Trappings were performed with battery-operated U.S. Centers for Disease Control and Prevention (CDC) miniature light traps using fine gossamer collection bags (model #512; John W. Hock Co., Gainesville, FL, USA) at appropriate trapping sites close to human dwellings and animal barns. Dry ice was occasionally used as a CO_2_ bait.

### Geographical and weather data acquisition

Geographical data from trapping sites were recorded by a global positioning system (TomTom N.V.; Amsterdam, the Netherlands). Hourly temperature and relative humidity data for trapping regions were retrospectively obtained from the Central Institute for Meteorology and Geodynamics (ZAMG). Together with the sand fly findings obtained in this study, published trapping sites were georeferenced into a distribution map using QGIS 3.4.11 [14].

### Morphological identification

Head and terminal segments of the abdomen of all caught sand fly specimens were dissected and mounted on a glass slide in CMCP-10 high-viscosity mountant (Polysciences Europe GmbH, Hirschberg an der Bergstrasse, Germany). Identification was based on morphological parameters of the male genitalia, the female spermatheca and the pharyngeal armature [15]. Additionally, fluorescence microscopy was used (NIKON Eclipse E 800; Nikon Instruments, Amstelveen, the Netherlands) to detect and identify the hardly visible female spermatheca as this structure can be illuminated by autofluorescence under UV light at a wave length of 330–380 nm.

### Molecular identification

DNA was isolated from the remaining bodies with QIAamp® DNA Mini Kit 250 (QIAGEN, Hilden, Germany). For species identification, a 658-bp fragment of the cytochrome *c* oxidase subunit I gene (*coxI*) was PCR-amplified following the protocol of Folmer et al. [16] using primers LCO-1490 and a newly designed reverse primer CoxUniEr (5′–AAA CTT CAG GGT GAC CAA AAA ATC–3′) because the initially used reverse primer [16] did not deliver satisfying PCR results in this case. Confirmation was obtained by amplifying a 652-bp segment of the cytochrome *b* gene (*cytb*) and the neighboring tRNA-Ser gene using the newly designed primers CytbEf1 (5′–CAA TGA ATT TGA GGA GGA TTT GT–3′) and CytbEr2 (5′–CTA TCT AAT GTT TTC AAA ACA ATT G–3′). The oligonucleotide sequence calculator OligoCalc was used to calculate GC contents, melting temperatures and optimal primer lengths and to exclude self-complementarity (http://biotools.nubic.northwestern.edu/OligoCalc.html). Amplification by PCR was conducted in a reaction volume containing 10× reaction buffer B, 2.5 mM MgCl_2_, 1.6 mM dNTPs, 1 µM primers, 1.25 units DNA polymerase and 1–5 µl DNA; sterile H_2_O was added to a final volume of 50 µl. The gene fragment was amplified using the following conditions: 95 °C for 15 min, followed by 35 cycles of 95 °C for 1 min (denaturation), 52 °C for 1:30 min (annealing) and 72 °C for 2 min (elongation), followed by a final extension of 72 °C for 10 min.

All PCR amplifications were performed with an Eppendorf Mastercycler modular PCR system (Eppendorf AG, Hamburg, Germany). Bands were analyzed with a Gel Doc™ XR+ Imager (Bio-Rad Laboratories, Inc., Hercules, CA, USA), and cut out of the gel and purified with an Illustra™ GFX™ PCR DNA and Gel Purification kit (GE Healthcare, Buckinghamshire, UK). Sanger sequencing was performed with the Applied Biosystems SeqStudio Genetic Analyzer (Thermo Fisher Scientific, Waltham, MA, USA). Sequences were obtained from both strands, and a consensus sequence was generated using the DNA sequence analysis tool GeneDoc 2.7.0. Sequence identities were revealed by comparing obtained sequences to sequences available in the GenBank.

### Screening for *Leishmania* spp.

Female specimens were screened by PCR and amplifications performed as described above. The primers LITSR/L5.8S targeting the internal transcribed spacer 1 (ITS1) gene were used, following the PCR protocol of El Tai et al. [17].

### DNA sequence analyses

Available sequences for comparison were downloaded from GenBank and aligned with the obtained sequences using ClustalX 2.1 for multiple alignment and GeneDoc 2.7.0. for manual editing and data analysis. DnaSP v.5 [18] was used to identify unique haplotypes. To assess genetic structure among groups, among populations and within populations, respectively, we calculated and visualized median joining networks [19] and analysis of molecular variance (AMOVA) with Popart v.1.7 [20]. For further clarification of species boundaries, pairwise distances and maximum likelihood (ML) analyses using unique haplotypes were calculated in MEGA X [21]. Based on best-fit evolutionary model selection, the Tamura’s 3-parameter model and Tamura–Nei’s parameter model with bootstrap support of 1000 replications were applied for *cox*I and *cytb*, respectively.

Results were compared to calculations of the Automatic Barcode Gap Discovery (ABGD) web-interface program (https://bioinfo.mnhn.fr/abi/public/abgd/), which generates Kimura-2-parameter (K2P) distances and assigns sequences to hypothetical species. Default settings of intraspecific divergence (*P*) of 0.001–0.1 were applied [22].

All sequence data were submitted to GenBank; barcodes, collection details and voucher material were deposited with ABOL and BOLD.

## Results

### Entomological survey

**Fig. 1:**
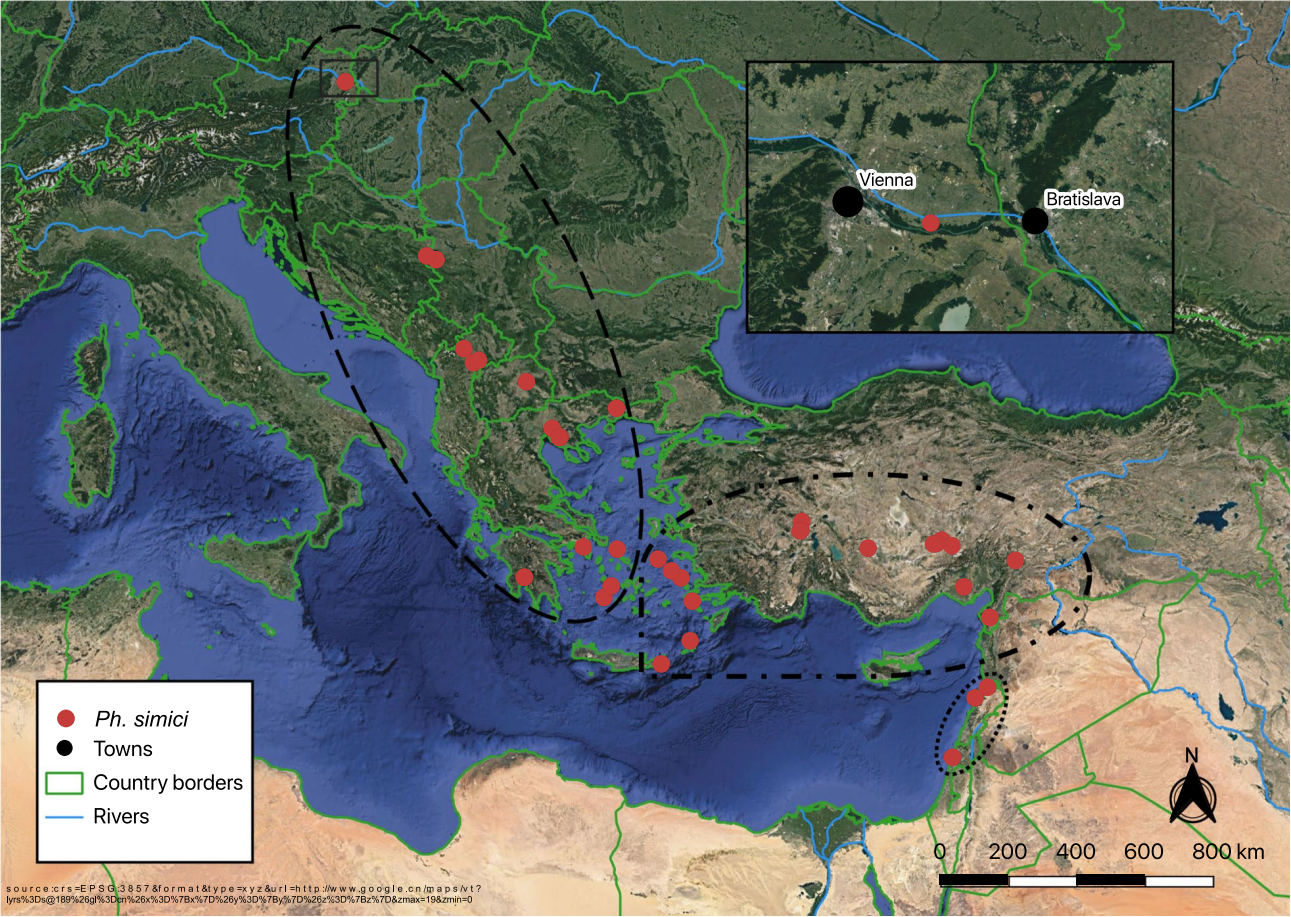
*Phlebotomus simici* distribution map, including the first finding in Austria. All published *Ph. simici* records with available coordinates were included. The three *Ph. simici* lineages suggested by the analysis of molecular variance (AMOVA), namely Europe, Turkey and Israel, are enclosed in dashed, dash–dotted and dotted lines, respectively. For some of the georeferenced *Ph. simici* records no sequence data are available

Inspection of insects caught in the field revealed, as in previous studies, *Phlebotomus mascittii*, in very low numbers, but also a single female specimen of *Phlebotomus simici* NITZULESCU, 1931, namely from Orth an der Donau (48.14462411 latitude, 16.69736534 longitude) in the night of 8–9 July at a local farm (Fig. 1). The CDC light trap baited with dry ice had been put up at the property in a barn with a natural floor used for hay storage. Several animals, including a dog, cats, chicken, geese, goats, pigs and rabbits, were kept at the property. The mean night temperature and mean relative humidity (RH) were 15.6 °C and 62.3%, respectively, in the trap night of 9 July. On 10 and 11 July, when no sand flies were in the traps, the mean night temperature was 15.5 °C and 13.2 °C, respectively, and the mean RH was 53.4 and 71.4%, respectively. The village is located in the federal state of Lower Austria in the eastern part of Austria directly along the River Danube, approximately 15 km west of Vienna. The annual mean temperature in Orth an der Donau is 9.9 °C and the annual mean precipitation is 627 mm.

### Species identification

**Fig. 2:**
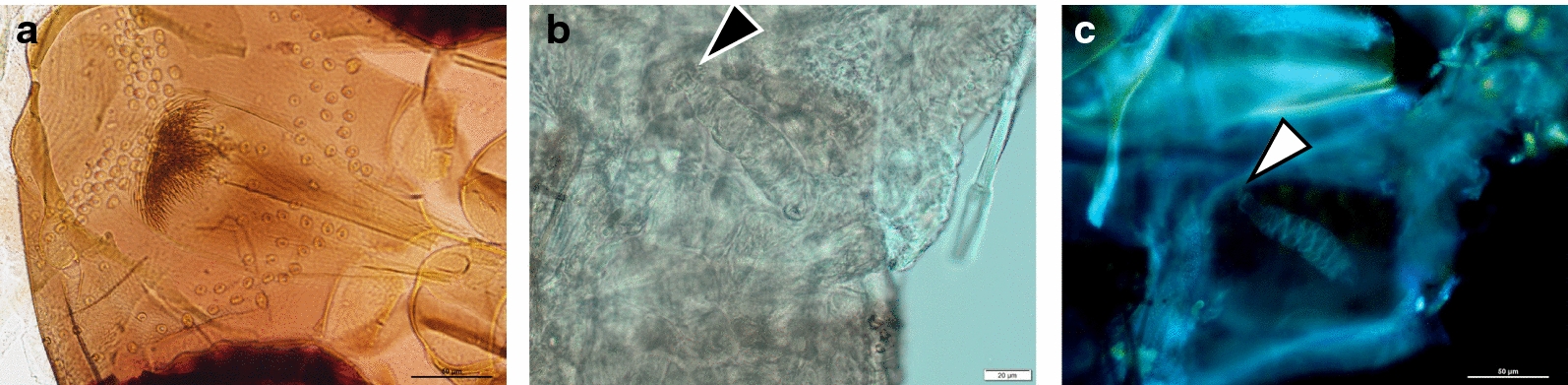
Morphological identification of *Ph. simici*. Pharynx (**a**), spermatheca (**b**), and autofluorescent spermatheca under UV light (**c**). Arrowhead in** b** and** c** indicates the tip of the spermatheca and the missing neck, respectively, typical characters for *Adlerius*

The specimen was morphologically identified by characters of the pharynx and spermatheca as belonging to the subgenus *Adlerius* NITZULESCU (Fig. 2). The obtained *cox*I sequence (GenBank: MN812831.1) was queried against available sequences in GenBank by BLAST and identified as *Ph. simici* NITZULESCU, 1931 [23]. Sequence identity ranged from 95.99 to 99.85% compared to sequences of *Ph. simici* originating from Turkey (MN086700.1) and Greece (KU519497.1), respectively. BLAST analysis of the obtained *cytb* sequence (GenBank: MN812836.1) confirmed species identification and showed 95.0–100% sequence identity with sequences of specimens from Crete, Greece (GenBank: MT452061.1) and North Macedonia (GenBank: MT452053.1), respectively. No *Leishmania* spp. DNA was detected in any of the sand flies by PCR analysis.

### Haplotype analysis of *Ph. simici* based on *cox*I sequences

Sequences of *Ph. simici* available in GenBank were edited to compile a dataset of 51 *coxI* sequences with a length of 551 bp without gaps and stop codons for haplotype analysis (Table 1). Forty haplotypes were identified, defined by 53 variable sites, of which 36 were parsimony informative, with an overall haplotype diversity (Hd) of 0.985 and an overall nucleotide diversity (π) of 0.229.

**Table 1:** Data on all *Phlebotomus simici* specimens included in the haplotype network analysis based on *coxI* and *cytb* sequences

Region	*coxI*		*cytb*		Reference
	GenBank	Haplotype	GenBank	Haplotype	
Austria	MN812831.1	Hap_1	MN812836.1	Hap_1	Present study
North Macedonia	MT452050.1, MT452051.1	Hap_2, Hap_3	MT452052.1, MT452053.1	Hap_1, Hap_2	Stefanovski et al. (GenBank)
Greece, Thessaloniki	KU519497.1–KU519500.1	Hap_3 Hap_7	–	–	Chaskopoulou et al. [26]
Greece, Peloponnese	MT452054.1–MT452056.1	Hap_8, Hap_9	MT452057.1–MT452059.1	Hap_3–Hap_5	Chaskopoulou et al. (GenBank)
Greece, Crete	MT452060.1	Hap_10	MT452061.1	Hap_6	Antoniou et al. (GenBank)
Turkey	MN086690.1–MN086717.1	Hap_11–Hap_38	–	–	Kasap et al. [28]
Israel	KX822734.1, KX822735.1	Hap_39, Hap_40	–	–	Akad et al. (GenBank)

*coxI*, cytochrome *c* oxidase subunit I gene;* cytb*, cytochrome* b* gene

**Fig. 3:**
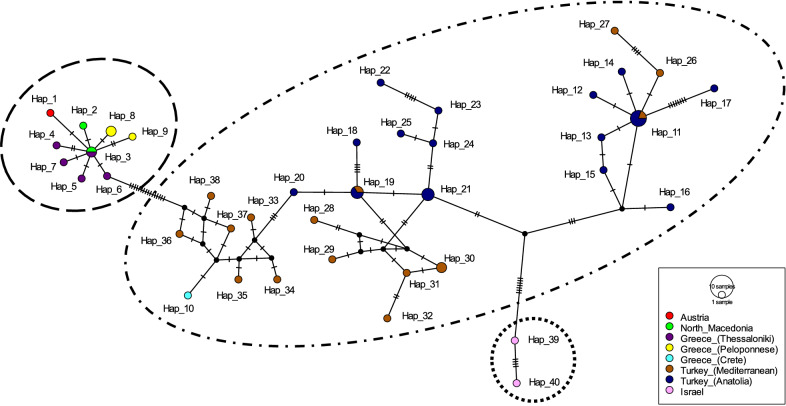
Haplotype (*Hap*) network of *Ph. simici* based on cytochrome *c* oxidase subunit I (*coxI*) sequences. The three *Ph. simici* lineages suggested by AMOVA, namely Europe, Turkey and Israel, are enclosed in dashed, dash-dotted and dotted lines, respectively

The haplotype of the Austrian *Ph. simici* specimen (Hap_1) clustered within a conserved European group that included haplotypes of specimens from North Macedonia (Hap_2, Hap_3), Thessaloniki, Greece (Hap_3–Hap_7) and Peloponnese, Greece (Hap_8, Hap_9). The haplotype from a specimen originating from Crete, Greece (Hap_10) clustered within the haplotypes of specimens originating from Turkey (Hap_11–Hap_38). A small third group was observed, consisting of both haplotypes of specimens from Israel (Hap_39, Hap_40) (Fig. 3). Analysis of molecular variance revealed 85.6% genetic variation between the three groups, and the comparably large genetic distance between the groups was supported by a high F_ST_ value (Table 2).

**Table 2:** Analysis of molecular variance of 51 *Ph. simici* individuals based on *coxI* sequences

Variance	*df*	Sum of squares	*σ*^2^	Percentage variance	Statistics	*P* value
Among groups	2	3618.423	174.418	85.63431	F_ST_ = 0.87663	< 0.001
Among populations	5	237.54	4.131	2.02828	F_SC_ = 0.14119	0.003
Within populations	43	1080.527	25.129	12.33741	F_CT_ = 0.85634	< 0.001
Total	50	4936.49	203.678	100		

*df*, Degrees of freedome; F_ST_, fixation index

### Haplotype analysis of *Ph. simici* based on *cytb* sequences

Altogether, seven sequences with a length of 609 bp were included in the analysis (Table 1). Six haplotypes were identified, defined by 34 variable sites, of which six were parsimony informative with an overall Hd of 0.952 and an overall π of 0.322.

**Fig. 4:**
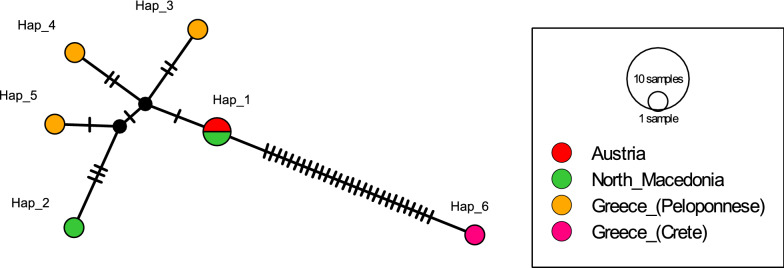
Haplotype network of *Ph. simici* based on *cytb* sequences

The sequence of *Ph. simici* from Austria was of the same haplotype (Hap_1) as a *Ph. simici* specimen from North Macedonia, both clustering with the haplotypes of other *Ph. simici* specimens from North Macedonia (Hap_2) and Peloponnese, Greece (Hap_3–Hap_5). The haplotype from a specimen from Crete, Greece (Hap_6) was clearly separated from all other haplotypes (Fig. 4). As the availability of *cytb* sequences was limited, AMOVA calculation was redundant.

### Pairwise sequence comparisons of *Adlerius* species

Altogether, 82 *coxI* sequences of *Ph. simici* and eight other species of the *Adlerius* subgenus with a length of 551 bp were included in the analysis (Additional file 1: Table S1). Pairwise distances (Pd) ranged from 0 to 18.1%. Hap_1 (Austria) showed the lowest distance (Pd 0.18%) to Hap_3, which is shared by specimens from North Macedonia and Thessaloniki, Greece, which further corroborated the clustering of the Austrian specimen within the European group in the haplotype network (Additional file 2: Table S2).

When sequences were grouped by species, calculated intraspecific mean distances ranged from 0.1 to 2.4%, the highest being calculated for *Ph. simici* (Table 3)*.* After a further division into three lineages, namely Europe, Turkey and Israel, mean intraspecific distances were 0.4, 1.5 and 0.7%, respectively.

**Table 3:** Interspecific mean *cox*I genetic distances (%) based on Tamura’s 3-parameter model

	Species analyzed	1	2	3	4	5	6	7	8	9	10
1	*Phebotomus simici*	*2.4*^*a*^									
2	*Phebotomus brevis*	5.3^a^	*0.9*								
3	*Adlerius* sp*.* Turkey	6.1^a^	3.9^a^	*0.8*							
4	*Adlerius* sp. Armenia	5.7^a^	3.6^a^	0.8^a^	*0.1*						
5	*Phebotomus balcanicus*	14.2	13.4	14.3	14.1	*3.7*^*a*^					
6	*Phebotomus halepensis*	14.5	12.3	13.1	13.0	8.6^a^	*1.4*				
7	*Phebotomus kyreniae*	14.8	13.5	14.9	14.6	7.1^a^	9.2^a^	*0.5*			
8	*Phebotomus chinensis*	15.7	15.8	15.5	15.4	13.6	13.1	14.8	*0.5*		
9	*Phebotomus longiductus*	16.8	15.8	16.1	15.2	13.6	12.4	14.1	14.5	*0.2*	
10	*Phebotomus arabicus*	17.3	15.4	15.8	15.5	11.2	8.1^a^	12.3	15.1	13.9	–^*b*^

Values in italics along the diagonal are intraspecific mean distances

^a^Indicates small interspecific distance or large intraspecific distance

^b^Only one sequence available

Interspecific mean distances between species ranged from 0.8 to 17.3%. While interspecific distances were low between *Ph. simici* and *Ph. brevis* THEODOR & MESGHALI, 1964, as well as between *Ph. simici* and an unknown *Adlerius* species from Turkey and Armenia (5.3–6.1%), they were high between *Ph. simici* and other *Adlerius* species (14.2–17.3%) included in the analyses. Mean distances between *Ph. simici* lineages ranged from 2.5 to 3.9%, and from 4.5 to 6.4% between *Ph. simici* groups, *Ph. brevis* and *Adlerius* spp. from Turkey and Armenia (Table 3). The lowest interspecific mean distance of 0.8% was observed between *Adlerius* specimens from Turkey and Armenia, clearly indicating that these two belong to the same unidentified species.

Nineteen *cytb* sequences with a length of 609 bp of specimens belonging to the subgenus *Adlerius* were included in the analysis (Additional file 3: Table S3). Pairwise distances ranged from 0 to 17.7%. The sequence of *Ph. simici* from Austria was 100% identical to that of a *Ph. simici* specimen from North Macedonia; pairwise distances to other *Ph. simici* sequences ranged from 0.5 to 4.9%, of which the highest was observed to *Ph. simici* from Crete, Greece (Additional file 4: Table S4).

Intraspecific mean distances were calculated for *Ph. simici* (1.9%), *Ph. halepensis* THEODOR, 1948 (1.0%) and *Ph. chinensis* NITZULESCU, 1931 (2.7%), as only one sequence of *Ph. brevis* was available (Table 4). After splitting *Ph. simici* into a European lineage and a Turkish lineage that included the specimen from Crete, the intraspecific mean distances within the European *Ph. simici* lineage was 0.6%.

**Table 4:** Interspecific mean *cytb* genetic distances (%) based on Tamura–Nei’s model

	Species	1	2	3	4
1	*Phebotomus simici*	*1.9*			
2	*Phebotomus brevis*	9.0	–^*a*^		
3	*Phebotomus halepensis*	13.5	13.7	*1.0*	
4	*Phebotomus chinensis*	15.4	15.0	15.5	*2.7*^*b*^

Values in italics along the diagonal are intraspecific mean distances

^a^Only one sequence available

^b^Indicates small interspecific distance or large intraspecific distance

Interspecific mean distances ranged from 9.0% between *Ph. simici* and *Ph. brevis* to 15.5% between *Ph. halepensis* and *Ph. chinensis* (Table 4). After splitting *Ph. simici* into a European and a Turkish lineage (including the specimen from Crete), interspecific mean distances were 5.1% between the two groups, 9.0% between *Ph. simici* European lineage and *Ph. brevis* and 9.3% between *Ph. simici* Turkey lineage and *Ph. brevis*.

### Maximum likelihood analysis of *cox*I

**Fig. 5:**
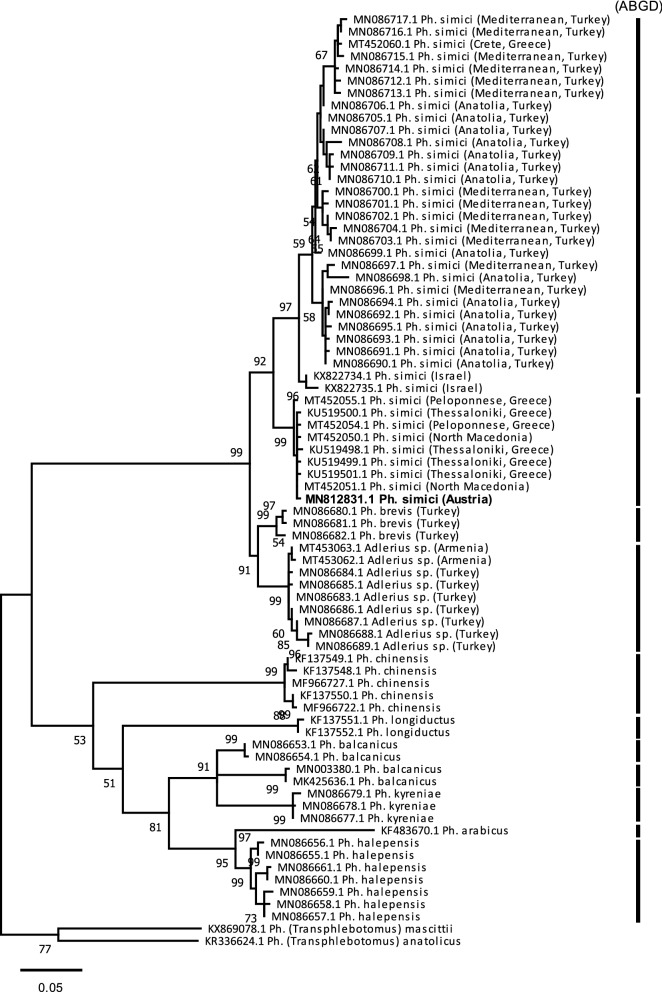
Maximum likelihood (ML) tree calculated based on *cox*I sequences of *Adlerius* spp. *Ph*. (*Transphlebotomus*) *mascittii* and *Ph*. (*Transphlebotomus*) *anatolicus* were used as outgroups. Vertical bars represent hypothetical species calculated by Automatic Barcode Gap Discovery (ABGD). Bootstrap values of > 50% are shown

The 82 sequences used for pairwise distance calculations showed 74 unique haplotypes, which were used for ML analysis. *Phlebotomus (Transphlebotomus) mascittii* GRASSI, 1908 and *Phlebotomus* (*Transphlebotomus*) *anatolicus* KASAP, DEPAQUIT & ALTEN, 2015, as well as *Phlebotomus neglectus* and *Phlebotomus perfiliewi* PARROT, 1930, were used as outgroups in two different approaches, respectively. In both approaches, two well-supported major clades were observed, clade 1 comprised *Ph. simici*, *Ph. brevis*, and an unidentified *Adlerius* species from Turkey and Armenia. Clade 2 comprised all other *Adlerius* species, namely *Ph. chinensis*, *Ph. longiductus* PARROT, 1928, *Ph. balcanicus* THEODOR, 1948, *Ph. arabicus* THEODOR, 1953, *Ph. kyreniae* THEODOR, 1958, and *Ph. halepensis* (Fig. 5, Additional file 5: Fig S1). Calculations resulted in three well-supported lineages of *Ph. simici* that matched the clustering of the median-joining network. An intraspecific threshold value of 0.7% was used for ABGD analysis, which partitioned the sequences into 11 groups. Calculations were in concordance with ML, with one exception, *Ph. simici* was split into two hypothetical species, namely Turkey + Israel and Europe. The unknown *Adlerius* sp. specimens from Turkey and Armenia were shown to belong to one single species and were identified as a sister species of *Ph. brevis* and together forming the sister group of *Ph. simici* (Fig. 5).

### Maximum likelihood analysis of *cytb*

**Fig. 6:**
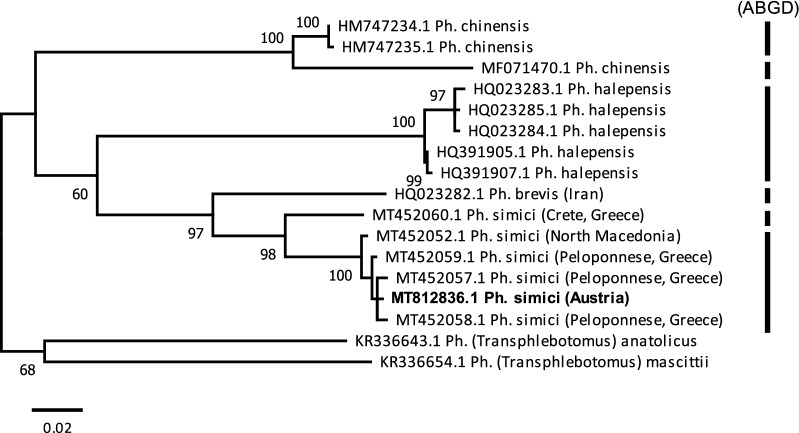
ML tree calculated based on *cytb* sequences of *Adlerius* spp. *Ph*. (*Transphlebotomus*) *mascittii* and *Ph*. (*Transphlebotomus*) *anatolicus* were used as outgroups. Vertical bars represent hypothetical species calculated by ABGD. Bootstrap values of > 50% are shown

The 19 sequences used for pairwise distance calculations showed 15 unique haplotypes, which were used for ML analysis. *Phlebotomus mascittii* and *Ph. anatolicus* as well as *Ph. neglectus* and *Ph. perfiliewi* were used as outgroups in two different approaches. In both approaches, two well-supported major clades were observed, clades 1 and 2, respectively. Clade 1 comprised *Ph. simici*, *Ph. brevis* and *Ph. halepensis*, which further corroborated that *Ph. simici* and *Ph. brevis* are sister species. Clade 2 comprised *Ph. chinensis*, which was split into two lineages. An intraspecific threshold value of 1.29% was used for the Automatic Barcode Gap Discovery (ABGD) analysis, which partitioned the sequences into six groups. ABGD grouped all four species as different groups with two exceptions: (i) *Ph. chinensis* was split into two lineages and (ii) *Ph. simici* from Crete, Greece was computed as a unique *Ph. simici* lineage. These results are in concordance with those of the ML analysis (Fig. 6; Additional file 6: Fig. S2).

## Discussion

This study reports the first finding of *Phlebotomus simici* in Austria, which is the northern- and westernmost record of this species to date, highlighting further the necessity of more detailed sand fly research in Austria and Central Europe in general. With the exception of prior *Ph. mascittii* findings in eastern parts of Austria, the sand fly fauna has remained unexplored and probably underreported in this country [5, 6].

The observation of *Ph. simici* in Austria is rather unexpected, as this species has never been reported in any of the countries bordering Austria (Table 5). Prior to this study, only a single species, namely *Ph. mascittii*, had been recorded in Austria, similar to neighboring Slovakia. In Austria’s neighboring countries that are known to harbor more than one species of sand flies, various sand fly species have been recorded, such as *Ph. mascittii* and *Ph. perniciosus* in Germany and *Ph. mascittii*, *Ph. perfiliewi*, *Ph. neglectus* and *Ph. papatasi* SCOPOLI, 1786 in Hungary, but never *Ph. simici*. Even in the countries bordering Austria to the south (Italy and Slovenia), both of which have a relatively diverse sand fly fauna comprising several different species, *Ph. simici* has never recorded. Geographically, the closest recent records of *Ph. simici* are from Serbia, which borders Hungary, one of Austria’s direct neighbors to the east, to the south.

**Table 5:** Checklist of reported sand fly species in Austria and its neighboring countries

Country	Species	Reference	GenBank *cox*I
Austria	*Phlebotomus* (*Adlerius*) *simici* NITZULESCU, 1931	Present study	Yes
	*Phlebotomus* (*Transphlebotomus*) *mascittii* GRASSI, 1908	Naucke et al. [5], Poeppl et al. [6]	Yes
Czech Republic	None observed	–	–
Germany	*Phlebotomus mascittii*	Oerther et al. [38]	No
	*Phlebotomus* (*Laroussius*) *perniciosus* NEWSTEAD, 1911	Naucke et al. [3]	No
Hungary	*Phlebotomus mascittii*	Trájer et al. [10]	No
	*Phlebotomus (Phlebotomus) papatasi* SCOPOLI, 1786		No
	*Phlebotomus (Laroussius) neglectus* TONNOIR, 1921		No
	*Phlebotomus (Laroussius) perfiliewi* PARROT, 1930		No
Italy	*Phlebotomus mascittii*	Dantas-Torres et al. [46]	No
	*Phlebotomus perniciosus*		No
	*Phlebotomuspapatasi*		No
	*Phlebotomusneglectus*		No
	*Phlebotomusperfiliewi*		Yes
	*Phlebotomus* (*Laroussius*) *ariasi* TONNOIR, 1921		No
	*Phlebotomus* (*Paraphlebotomus*) *sergenti* PARROT, 1917		No
	*Sergentomyia (Sergentomyia) minuta* RONDANI, 1843		No
Liechtenstein	None observed	–	–
Slovakia	*Phlebotomus mascittii*	Dvořák et al. [8]	Yes
Slovenia	*Phlebotomus mascittii*	Praprotnik [47]	Yes
	*Phlebotomus perniciosus*	Ivović et al. [48]	No
	*Phlebotomus papatasi*		No
	*Phlebotomus neglectus*		No
	*Sergentomyia minuta*		No
Switzerland	*Phlebotomus mascittii*	Knechtli and Jenni [49]	No
	*Phlebotomus perniciosus*		No
	*Sergentomyiaminuta*		No

Subgenus, author and year of description are provided at first mention of the respective species. Countries are presented in alphabetical order

*Ph. simici* belongs to the *Adlerius* NITZULESCU subgenus, which includes around 20 described as well as several undescribed species with predominantly Eurasian distribution and an assumed origin in Central Asia [24]. *Ph. simici* is frequently reported in Balkan [25–28] and Middle Eastern countries [29, 30]. Recent reports from North Macedonia (V. Dvořák, verbal communication), Kosovo [31] and Serbia [32] point towards a northward European distribution, which is further corroborated by an older mention from Croatia [33]. *Ph. simici* is also mentioned in an ex-Yugoslavian study, but it is not entirely clear whether it was indeed recorded in areas which today belong to Croatia [34].

The periurban village where the *Ph. simici* specimen was caught in Austria is located in the Danube valley in the very eastern part of the country, which is one of the the warmest parts of Austria. Microclimatic conditions in river valleys support the establishment and prevalence of local populations of sand flies north of the core area of European distributions, as shown by the occurrence of *Ph. mascittii* in the Rhine valley [35]. The Danube valley has been assumed to be particularly suitable for sand fly occurrence [36]. The sampling location exhibits perfect breeding site requirements for sand flies, having several buildings with natural floors and various animal hosts, including a dog, poultry, swine, rabbits and goats, close to human dwellings. Typically, *Ph. mascittii* is also found at similar locations in Central Europe [2, 5, 6, 8], which raises the question if possibly these two species also overlap in other regions and whether more *Ph. simici* populations are already established but have been overlooked in the past.

That only a single specimen was detected may be attributed to several factors. Firstly, even though July is usually the warmest month in Austria, abnormal weather conditions were observed in 2019, with great temperature fluctuations. The night temperature was only 15.6 °C in the trapping night and decreased in the consecutive nights, probably temporarily suspending sand fly activity. In Romania, *Ph. perfiliewi* was observed to be active at 15 °C minimum night temperature, but no activity has been observed below this temperature [37]. Secondly, this finding supposedly represents the northern distribution limit of this species and, therefore, low population densities and consequently small trapping numbers are to be expected. In Austria, trapping rates are generally extremely low, also for *Ph. mascittii*, with typical trapping numbers of < 5 specimens per night [5, 6, 38]. In Slovakia, only a single specimen of *Ph. mascittii* has ever been trapped, namely in 2016 [6, 8]. A comparative study by Obwaller et al. [7] reported huge differences in the numbers of trapped *Ph. mascittii* specimens in consecutive years at two locations in Austria. These observations suggest that sand fly activity—and thus trapping success—might not only depend on temperature but that other factors may also play a role.

Identification of the female specimen was challenging, and morphological identification was only possible to the subgenus level. Both, pharynx and spermatheca, showed typical *Adlerius* structures; however, the spermatheca was barely visible by light microscopy. An additional assessment of the spermatheca under UV light illuminated structures that confirmed subgenus *Adlerius*. To our knowledge, this is the first report of the use of autoimmunofluorescence for sand fly identification. The application of this technique might add a valuable tool for use in the morphological examination of spermathecae. While its suitability for identification to the species level has to be further evaluated, it clearly contributes to the visualization of the otherwise often hardly visible spermatheca. The impossibility of identifying the female specimen based on morphology to the species level is not surprising; *Adlerius* females are often unidentifiable by morphology. This is particularly known for *Ph. simici* and *Ph. brevis*, two species that overlap in all morphological characters used to distinguish females of the subgenus *Adlerius* [24]. For example, Perrotey et al. [30] reported that females of sympatrically occurring *Ph. simici* and *Ph. brevis* in Lebanon were undistinguishable on the basis of morphological characters.

To clarify conflicting morphological identifications, molecular approaches using suitable marker genes are needed. In our study, species identification was possible by sequencing the *coxI* gene, a classical DNA barcoding marker. Interestingly, sequence identity ranged from 95.99 to 99.85%, to sequences of *Ph. simici* from Turkey and Greece, respectively. Further sequence analyses revealed a monophyletic group of three distinct lineages of *Ph. simici*; however, mean pairwise distances between the three lineages were unexpectedly high for within one species. In addition, interspecific distances to *Ph. brevis* and the unidentified *Adlerius* species from Turkey and Armenia were rather low (< 10%) compared to distances to the other *Adlerius* species (> 10%) included in the analyses. This finding indicates that *Ph. simici*, *Ph. brevis* and other *Adlerius* spp. are genetically very close and that *coxI* might not be an ideal genetic marker for such closely related species.

*CoxI *has been a commonly used genetic marker for species identification since its introduction as “the barcoding gene” by Hebert et al. [39] and, consequently, sequence availability in GenBank is high and *coxI* is frequently used for sand fly identification and interspecific comparisons [40]. Although there is no common cut-off value for species delimitation, Hebert et al. [39] observed a mean divergence value of 11.3% between species and only a small fraction showed ≤ 2% divergence. However, in this study we observed pairwise distances between *Ph. simici*, *Ph. brevis* and another *Adlerius* sp. that was far less than 10%. In particular, the mean interspecific distance between *Ph. simici* and *Ph. brevis* was only marginally higher than the mean interspecific distances between the three observed *Ph. simici* lineages, clearly indicating that *Ph. simici*, *Ph. brevis* and the as yet unidentified *Adlerius* species have a short history of divergence and are thus challenging to differentiate by *coxI* sequences. In contrast, interspecific distances of *Ph. simici* to further *Adlerius* specimens included in the analysis far exceeded 10% and, therefore, these species are easy to separate.

To corroborate our results, *cytb* was used as a second genetic marker, even though sequence availability is rather poor for *Adlerius* species. *Cytb* is the most commonly used genetic marker in sand fly systematics [40]. Further confirmation of species delimitation was achieved by comparing the obtained *Ph. simici* sequences with reference sequences of *Ph. simici* and *Ph. brevis* from Iran. Although the intraspecific distance within *Ph. simici* was similarly high as that observed for *coxI*, the calculated interspecific distance was almost double the mean interspecific distance of *coxI* between *Ph. simici* and *Ph. brevis* (9.1%) and clearly separated these two species.

*Ph. simici* is an assumed but unproven vector species for *Leishmania infantum* [24]. Even though the specimen found tested negative for *Leishmania* DNA, this species has been shown to be highly anthropophilic [26], which is important for its potential relevance in *Leishmania* transmission to humans.

Taken together, the finding of a single *Ph. simici* specimen in Austria does not allow any inference on deeper population genetic structures; however, interesting results at the sequence level were obtained and should be considered in future studies. It is obvious that a single specimen cannot prove the existence of a permanent population and does not provide any information on the actual population size. However, eastern parts of Austria in particular have been shown to be suitable for sand flies, which is underlined by continuous trappings of *Ph. mascittii*, the closest population being found in Rohrau, approximately 15 km away from the location reported in this study [5, 6]. As yet, the origin and routes of dispersal are still unclear. By finding a unique but genetically very close haplotype and a shared haplotype of *coxI* and *cytb*, respectively, to a haplotype from North Macedonia, post-glacial northward recolonization from this area seems likely. This is further corroborated by recent findings in Serbia [32]. Temperatures in Central Europe during the Holocene optimum around 6000 years ago were comparable to those of today, and the presence of Mediterranean species in Central Europe may result from northward recolonization events from different refugial areas at that time [41]. The known distribution of *Ph. simici* and the high interspecific distances between the European, Turkish and Israeli lineages suggest that *Ph. simici* is most certainly a polycentric Balkanopontomediterranean species. The split between the European and the Turkish *Ph. simici* lineages might have taken place during one of several complex paleogeographic events that separated the Aegean region into eastern and western parts, as has been demonstrated for the *Transphlebotomus* subgenus, where separation of the five species, including *Ph. mascittii*, was dated back to major biogeographic events in the Aegean region [42]. Inference on genetic divergence can be tricky, and high mutation rates based on molecular clock calibrations of 5.7%/Mya [43] and 19.2%/Mya [44] have been published at the population level compared to a commonly applied rate of 2.3%/Mya for mitochondrial DNA [45]. Thus, the clarification of separation events between *Ph. simici* lineages and between other *Adlerius* species should be the subject of further studies, including a more representative set of populations.

## Conclusions

Although the finding of only a single *Ph. simici* specimen is reported here, this study presents a unique and important finding for Austria and Central Europe in general. It clearly shows that current knowledge on sand fly distribution and species diversity is still scarce in Austria, but also in the larger area of Europe. Further entomological surveys are needed to elucidate the current distribution and species composition of sand flies, as well as to assess their epidemiological significance in Central Europe, especially in climatically favorable regions which may already be inhabited by overlooked populations of known and unknown species. This is of greatest importance, as a warming climate may lead to further growth of established sand fly populations and hence further dispersal. However, the increasing absence of traditional farms as commonly observed microhabitats for sand flies might have a limiting effect on future dispersal in Austria. Evaluation and sampling of other potential trapping sites should be attempted in future studies. Moreover, this study corroborates that morphological discrimination of sand fly species can be tricky or even impossible. The newly introduced approach that takes advantage of the autofluorescence of chitin might constitute a very valuable tool. Molecular identification techniques have limitations and should always be interpreted with caution, particularly for closely related or cryptic species. The inclusion of at least a second marker gene or technique is advised in these cases. Although precise dispersal routes from refugial areas to Central Europe remain unknown, phylogenetic analyses in this study shed light on the relationships within *Ph. simici* and between *Adlerius* species.

## Supplementary information

**Additional file 1: Table S1. **Included* coxI* sequences of* Adlerius* spp. for pairwise distance calculations.

**Additional file 2: Table S2. **Pairwise distances (%) of* coxI* based on Tamura’s 3-parameter model with 1000 bootstrap values. Description in brackets refer to the respective haplotype in the network. Standard errors are shown in blue.

**Additional file 3: Table S3. **Included* cytb* sequences of* Adlerius* spp. for pairwise distance calculations.

**Additional file 4: Table S4.** Pairwise distances (%) of* cytb* based on Tamura–Nei’s parameter model with 1000 bootstrap values. Description in brackets refer to the respective haplotype in the network. Standard errors are shown in blue.

**Additional file 5: Figure S1.** Maximum likelihood (ML) tree calculated based on *coxI *sequences of* Adlerius* spp.* Ph.* (*Laroussius*)* neglectus* and* Ph.* (*Laroussius*)* perfiliewi* were used as outgroup. Vertical bars represent hypothetical species calculated by ABGD. Bootstrap values > 50 % are shown.

**Additional file 6: Figure S2.** Maximum likelihood (ML) tree calculated based on* cytb* sequences of* Adlerius* spp.* Ph.* (*Laroussius*)* neglectus* and* Ph*. (*Laroussius*)* perfiliewi* were used as outgroup. Vertical bars represent hypothetical species calculated by ABGD. Bootstrap values > 50% are shown.

## Data Availability

All data generated and analyzed during this study was included in the article.

## Abbreviations

ABGD: Automatic Barcode Gap Discovery (program)
ABOL: Austrian Barcode of Life
BOLD: Barcode of Life Data System
*cox**I*: Cytochrome *c* oxidase subunit I gene
*cytb*: Cytochrome* b *gene
ML: Maximum likelihood
Mya: Million years
ZAMG: Central Institute for Meteorology and Geodynamics

## References

[1] Ready PD (2010). Leishmaniasis emergence in Europe. Eurosurveillance.

[2] Naucke TJ, Pesson B (2000). Presence of Phlebotomus (Transphlebotomus) mascittii Grassi, 1908 (Diptera: Psychodidae) in Germany. Parasitol Res..

[3] Naucke TJ, Schmitt C (2004). Is leishmaniasis becoming endemic in Germany?. Int J Med Microbiol..

[4] Depaquit J, Naucke TJ, Schmitt C, Ferté H, Léger N (2005). A molecular analysis of the subgenus Transphlebotomus Artemiev, 1984 (Phlebotomus, Diptera, Psychodidae) inferred from ND4 mtDNA with new northern records of Phlebotomus mascittii Grassi, 1908. Parasitol Res..

[5] Naucke TJ, Lorentz S, Rauchenwald F, Aspöck H (2011). Phlebotomus (Transphlebotomus) mascittii Grassi, 1908, in Carinthia: First record of the occurrence of sandflies in Austria (Diptera: Psychodidae: Phlebotominae). Parasitol Res.

[6] Poeppl W, Obwaller AG, Weiler M, Burgmann H, Mooseder G, Lorentz S (2013). Emergence of sandflies (Phlebotominae) in Austria, a Central European country. Parasitol Res..

[7] Obwaller AG, Poeppl W, Naucke TJ, Luksch U, Mooseder G, Aspöck H (2014). Stable populations of sandflies (Phlebotominae ) in Eastern Austria : a comparison of the trapping seasons 2012 and 2013. Trends Entomol..

[8] Dvořák V, Hlavackova K, Kocisova A, Volf P (2016). First record of Phlebotomus (Transphlebotomus) mascittii in Slovakia. Parasite..

[9] Farkas R, Tánczos B, Bongiorno G, Maroli M, Dereure J, Ready PD (2011). First surveys to investigate the presence of canine leishmaniasis and its phlebotomine vectors in Hungary. Vector-Borne Zoonotic Dis..

[10] Trájer AJ (2017). Checklist, distribution maps, bibliography of the Hungarian Phlebotomus (Diptera: Psychodidae) fauna complementing with the climate profile of the recent sandfly distribution areas in Hungary. Folia Faun Slovaca..

[11] Trájer AJ, Sebestyén V (2019). The changing distribution of Leishmania infantum Nicolle, 1908 and its Mediterranean sandfly vectors in the last 140 kys. Sci Rep..

[12] Beyreder J (1962). Ein Fall von Leishmaniose in Niederösterreich. Wien Med Wochenschr.

[13] Kollaritsch H, Emminger W, Zaunschirm A, Aspöck H (1989). Suspected autochthonous Kala-Azar in Austria. Lancet.

[14] QGIS Development Team. QGIS geographic information system. Open Source Geospatial Foundation Project. http://qgis.osgeo.org. 2019.

[15] Lewis DJ. A taxonomic review of the genus* Phlebotomus* (Diptera: Psychodidae). Bull Br Museum (Nat Hist). 1982;45:121–209.

[16] Folmer O, Black M, Hoeh W, Lutz R, Vrijenhoek R (1994). DNA primers for amplification of mitochondrial cytochrome c oxidase subunit I from diverse metazoan invertebrates. Mol Mar Biol Biotechnol..

[17] El Tai NO, Osman FO, El FM, Presber W, Schönian G (2000). Genetic heterogeneity of ribosomal internal transcribed spacer in clinical samples of Leishmania cfonovani spotted on filter paper as revealed by single-strand conformation polymorphisms and sequencing. Trans R Soc Trop Med Hyg..

[18] Librado P, Rozas J (2009). DnaSP v5: A software for comprehensive analysis of DNA polymorphism data. Bioinformatics.

[19] Bandelt H-J, Forster P, Röhl A (1999). Median-joining networks for inferring intraspecific phylogenies. Mol Biol Evol..

[20] Leigh JW, Bryant D. PopART: full-feature software for haplotype network construction. Methods Ecol Evol. 2015; 6:1110–6.

[21] Kumar S, Stecher G, Li M, Knyaz C, Tamura K (2018). MEGA X: Molecular Evolutionary Genetics Analysis across computing platforms. Mol Biol Evol..

[22] Puillandre N, Lambert A, Brouillet S, Achaz G (2012). ABGD, Automatic Barcode Gap Discovery for primary species delimitation. Mol Ecol..

[23] Nitzulescu V (1931). Essai de classification des phlébotomes. Annu Parasitol Hum Comp..

[24] Artemiev MM (1980). A revision of sandflies of the subgenus Adlerius (Diptera, Phlebotominae, Phlebotomus). Zool Zh..

[25] Christodoulou V, Antoniou M, Ntais P, Messaritakis I, Ivović V, Dedet J-P (2012). Re-emergence of visceral and cutaneous leishmaniasis in the Greek Island of Crete. Vector-Borne Zoonotic Dis..

[26] Chaskopoulou A, Giantsis IA, Demir S, Bon MC (2016). Species composition, activity patterns and blood meal analysis of sand fly populations (Diptera: Psychodidae) in the metropolitan region of Thessaloniki, an endemic focus of canine leishmaniasis. Acta Trop..

[27] Tsirigotakis N, Pavlou C, Christodoulou V, Dokianakis E, Kourouniotis C, Alten B (2018). Phlebotomine sand flies (Diptera: Psychodidae) in the Greek Aegean Islands: ecological approaches. Parasites Vectors..

[28] Kasap OE, Linton Y-M, Karakus M, Ozbel Y, Alten B (2019). Revision of the species composition and distribution of Turkish sand flies using DNA barcodes. Parasites Vectors..

[29] Svobodova M, Votypka J, Peckova J, Dvořák V, Nasereddin A, Baneth G (2006). Distinct transmission cycles of Leishmania tropica in 2 adjacent foci, northern Israel. Emerg Infect Dis..

[30] Perrotey S, Benabdennbi I, Haddad N, Pesson B, Leger N (2009). Electrophoretic and morphological differentiation between two sympatric species of Adlerius: Phlebotomus brevis and Phlebotomus simici (Diptera: Psychodidae). J Med Entomol..

[31] Vaselek S, Oguz G, Ayhan N, Ozbel Y, Kadriaj P, Ćupina AI (2020). Sandfly surveillance and investigation of Leishmania spp. DNA in sandflies in Kosovo. Med Vet Entomol..

[32] Vaselek S, Dvořák V, Hlavackova K, Ayhan N, Halada P, Oguz G (2019). A survey of sand flies (Diptera, Phlebotominae) along recurrent transit routes in Serbia. Acta Trop..

[33] Mulić R, Ustović AĆ, Ropac D, Tripković I, Stojanović D, Klišmanić Z (2009). Occurence of visceral and cutaneous leishmaniasis in Croatia. Mil Med..

[34] Simić Č, Kostić D, Nežić E, Živković V (1951). Prilog poznavanju flebotomina Jugoslavije. VI deo. Flebotomine Vojvodine, Bosne, Hercegovine, Dalmacije i Istre. Glas Srp Akad Nauk CCII, Odeljenje Med Nauk..

[35] Naucke TJ, Menn B, Massberg D, Lorentz S (2008). Sandflies and leishmaniasis in Germany. Parasitol Res..

[36] Aspöck H, Walochnik J (2009). When sandflies move north. Public Health..

[37] Cazan CD, Păstrav IR, Györke A, Oguz G, Alten B, Mihalca AD (2019). Seasonal dynamics of a population of Phlebotomus (Larroussius) perfiliewi Parrot, 1930 (Diptera: Psychodidae) in North-Eastern Romania. Parasitol Res..

[38] Oerther S, Jöst H, Heitmann A, Lühken R, Krüger A, Steinhausen I (2020). Phlebotomine sand flies in Southwest Germany: an update with records in new locations. Parasites Vectors..

[39] Hebert PDN, Ratnasingham S, de Waard JR (2003). Barcoding animal life: cytochrome c oxidase subunit 1 divergences among closely related species. Proc R Soc B Biol Sci..

[40] Depaquit J (2014). Molecular systematics applied to Phlebotomine sandflies: review and perspectives. Infect Genet Evol..

[41] Aspöck H (2008). Postglacial formation and fluctuations of the biodiversity of Central Europe in the light of climate change. Parasitol Res..

[42] Kasap OE, Dvořák V, Depaquit J, Alten B, Votypka J, Volf P (2015). Phylogeography of the subgenus Transphlebotomus Artemiev with description of two new species, Phlebotomus anatolicus n. sp. and Phlebotomus killicki n. sp.. Infect Genet Evol..

[43] Clarke TE, Levin DB, Kavanaugh DH, Reimchen TE (2001). Rapid evolution in the Nebria gregaria group (coleoptera: Carabidae) and the paleogeography of the Queen Charlotte islands. Evolution.

[44] Gratton P, Konopiński MK, Sbordoni V (2008). Pleistocene evolutionary history of the Clouded Apollo (Parnassius mnemosyne): Genetic signatures of climate cycles and a “time-dependent” mitochondrial substitution rate. Mol Ecol..

[45] Papadopoulou A, Anastasiou I, Vogler AP (2010). Revisiting the insect mitochondrial molecular clock: the mid-Aegean trench calibration. Mol Biol Evol..

[46] Dantas-Torres F, Tarallo VD, Otranto D (2014). Morphological keys for the identification of Italian phlebotomine sand flies (Diptera: Psychodidae: Phlebotominae). Parasites Vectors..

[47] Praprotnik E, Zupan S, Ivović V (2019). Morphological and molecular identification of Phlebotomus mascittii Grassi, 1908 populations from Slovenia. J Med Entomol..

[48] Ivović V, Kalan K, Zupan S, Bužan E (2015). Illegal waste sites as a potential micro foci of Mediterranean Leishmaniasis: first records of phlebotomine sand flies (Diptera: Psychodidae) from Slovenia. Acta Vet Brno..

[49] Knechtli R, Jenni L (1989). Distribution and relative density of three sandfly (Diptera: Phlebotominae) species in southern Switzerland. Ann Parasitol Hum Comparée..

