# Supplementary figures and images for: Phlebotomus (Adlerius) simici NITZULESCU, 1931: first record in Austria and phylogenetic relationship with other Adlerius species
Source: Parasit Vectors. 2021 Jan 6;14:20. doi: 10.1186/s13071-020-04482-8 (PMC7788815; doi:10.1186/s13071-020-04482-8)

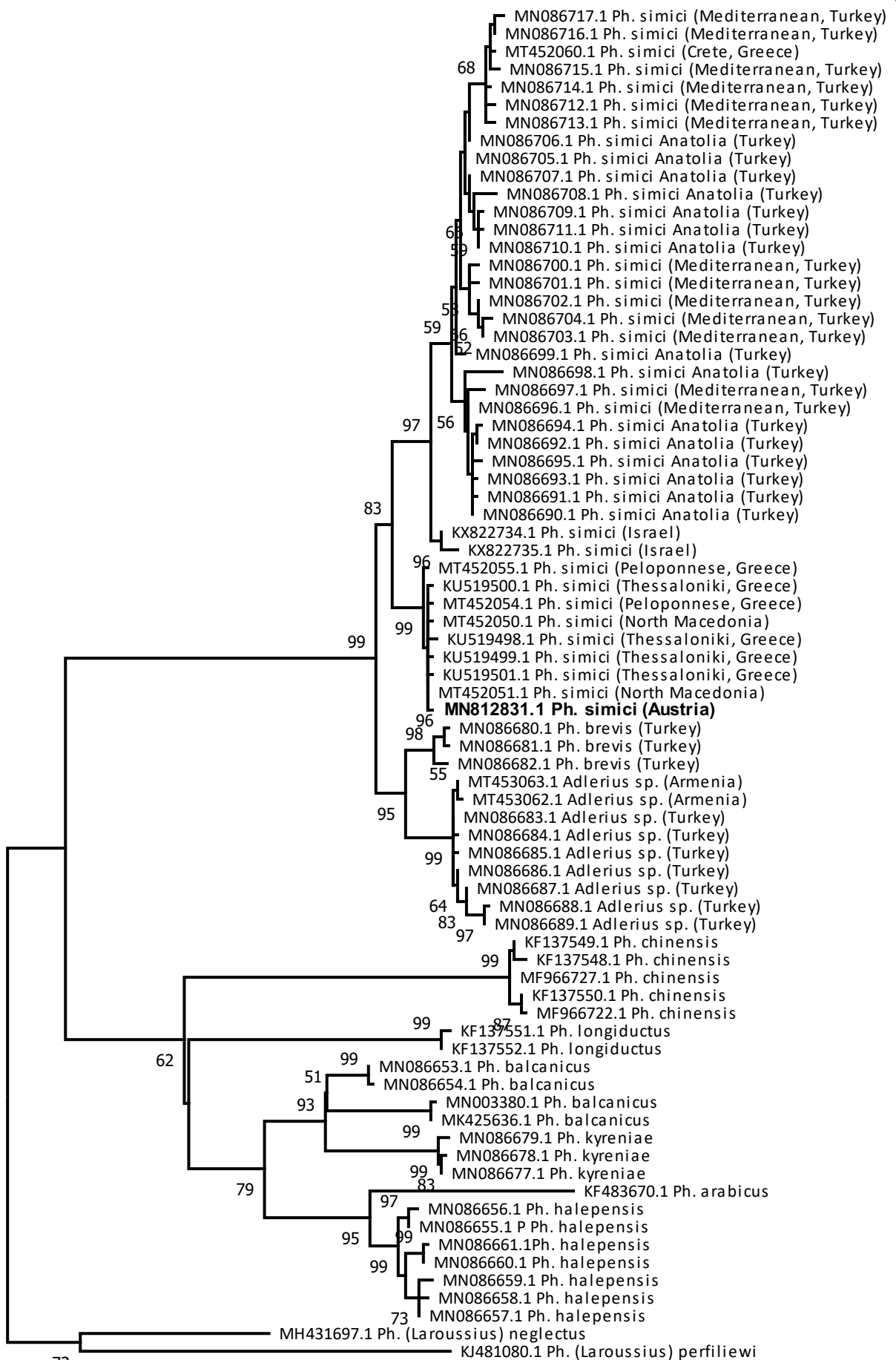

Supplement: Supplementary file 5 — Additional file 5: Figure S1. Maximum likelihood (ML) tree calculated based on coxI sequences of Adlerius spp. Ph. (Laroussius) neglectus and Ph. (Laroussius) perfiliewi were used as outgroup. Vertical bars represent hypothetical species calculated by ABGD. Bootstrap values > 50 % are shown. [file 13071_2020_4482_MOESM5_ESM.pdf]

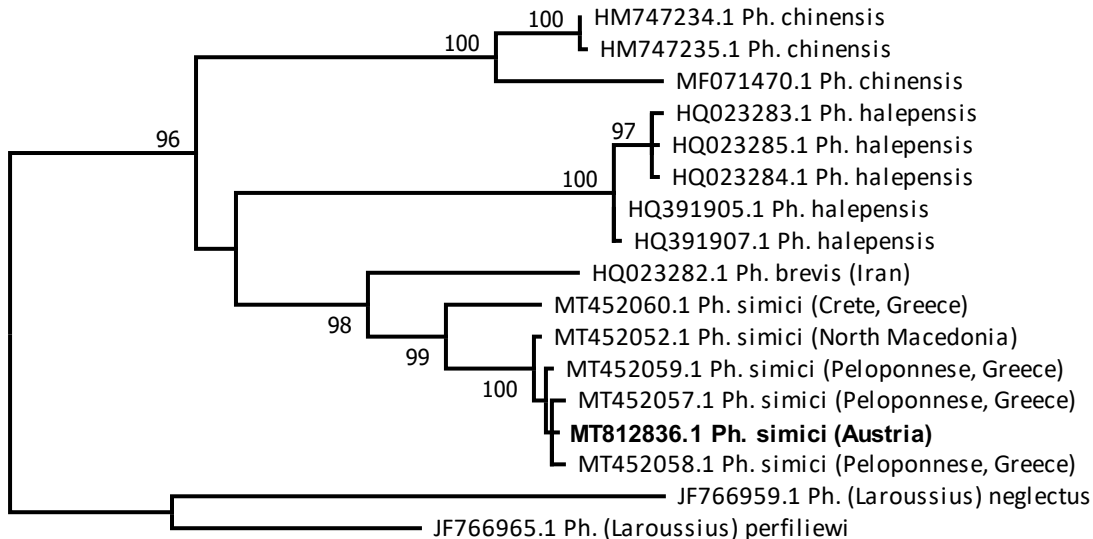

Supplement: Supplementary file 6 — Additional file 6: Figure S2. Maximum likelihood (ML) tree calculated based on cytb sequences of Adlerius spp. Ph. (Laroussius) neglectus and Ph. (Laroussius) perfiliewi were used as outgroup. Vertical bars represent hypothetical species calculated by ABGD. Bootstrap values > 50% are shown. [file 13071_2020_4482_MOESM6_ESM.pdf]
